# The Sialic Acid Binding Protein, Hsa, in *Streptococcus gordonii* DL1 also Mediates Intergeneric Coaggregation with *Veillonella* Species

**DOI:** 10.1371/journal.pone.0143898

**Published:** 2015-11-25

**Authors:** Peng Zhou, Jinman Liu, Xiaoli Li, Yukihiro Takahashi, Fengxia Qi

**Affiliations:** 1 Department of Microbiology and Immunology, College of Medicine, University of Oklahoma Health Sciences Center, Oklahoma City, Oklahoma 73104, United States of America; 2 Department of Oral Biology, College of Dentistry, University of Oklahoma Health Sciences Center, Oklahoma City, Oklahoma 73104, United States of America; 3 Department of Microbiology, School of Life Dentistry at Tokyo, Nippon Dental University, 1-9-20 Fujimi, Chiyoda-ku, Tokyo 102-8159, Japan; East Carolina University School of Medicine, UNITED STATES

## Abstract

Dental biofilm development involves initial colonization of the tooth’s surface by pioneer colonizers, followed by cell-cell coaggregation between the pioneer and later colonizers. *Streptococcus gordonii* is one of the pioneer colonizers. In addition to its role in oral biofilm development, *S*. *gordonii* also is a pathogen in infective endocarditis in susceptible humans. A surface adhesin, Hsa, has been shown to play a critical role in colonization of *S*. *gordonii* on the heart tissue; however, its role in oral biofilm development has not been reported. In this study we demonstrate that Hsa is essential for coaggregation between *S*. *gordonii* and *Veillonella* sp., which are bridging species connecting the pioneer colonizers to the late colonizers. Interestingly, the same domains shown to be required for Hsa binding to sialic acid on the human cell surface are also required for coaggregation with *Veillonella* sp. However, sialic acid appeared not to be required for this intergeneric coaggregation. This result suggests that although the same domains of Hsa are involved in binding to eukaryotic as well as *Veillonella* cells, the binding mechanism is different. The gene expression pattern of *hsa* was also studied and shown not to be induced by coaggregation with *Veillonella* sp.

## Introduction

It is believed that more than 700 bacterial species colonize the human oral cavity, and each human mouth may harbor as many as 120 species [1, 2]. The formation of this multispecies community involves a sequential process, with pioneer colonizers adhering to the tooth’s surface, followed by early/middle colonizers adhering to the pioneer colonizers, and finally by the late colonizers adhering to the early/middle colonizers [3, 4]. The mitis streptococci (*S*. *gordonii*, *S*. *oralis* and *S*. *sanguinis* etc.) are considered pioneer colonizers, which can comprise as much as 80% of the early dental biofilm population [5]. The *Veillonella* species are one of early/middle colonizers. They are closely associated with the streptococci via cell-cell coaggregation as well as metabolic complementation—lactic acid produced by the streptococci serves as major carbon source for veillonellae, and the consequential lactate removal raises local pH, thus relieving the streptococci from toxicity of their own metabolic waste. This mutualistic relationship helps *Veillonella* sp to colonize and grow in the early biofilm community, which then provides attachment sites and possibly different kinds of metabolic complementation for the late colonizers such as the periodontopathogen *Porphyromonas gingivalis* [6, 7]. Given the importance of early/middle colonizers such as veillonellae in biofilm development and dysbiosis, understanding the mechanism of coaggregation between *Veillonella* species and streptococci would provide an important knowledge base for developing strategies of disease prevention.

Our previous studies have identified a surface protein named Hag1 in *V*. *atypica* strain OK5, which is responsible for *Veillonella* binding to *S*. *gordonii* [8]. We further demonstrated that the binding partner for Hag1 is likely a protein, as proteinase treatment of *S*. *gordonii* completely abolished coaggregation [8]. In this study, we report the identification of the corresponding adhesin from *S*. *gordonii*. We show that a previously characterized sialic acid binding adhesin, Hsa, is required for coaggregation with *V*. *atypica* as well as other *Veillonella* species. We further demonstrate that the same functional domains of Hsa responsible for binding to sialic acid on mammalian cells also are required for coaggregation with *V*. *atypica*; however, a different mechanism seems to be utilized in Hsa binding to mammalian cells rather than to veillonellae cells. Finally, reporter expression analyses showed that *hsa* gene expression is not induced or enhanced by coaggregation with *V*. *atypica*.

## Materials and Methods

### Bacterial strains and culture conditions

Bacterial strains and plasmids used or constructed in this study are listed in Table 1. *Streptococcus gordonii* DL1 was grown in brain heart infusion broth (BHI; Difco), or on BHI agar plates. For transformant selection, cells were grown in Todd–Hewitt broth (Difco) with 0.3% yeast extract (Difco) (THYE) plus erythromycin (12.5 μg ml^-1^) or spectinomycin (500 μg ml^-1^). For selection of genetically complemented mutant strains, THYE broth supplemented with 250 μg ml^-1^ spectinomycin was utilized. *Veillonella atypica* OK5 and other veillonella strains were grown in brain heart infusion broth (BHI; Difco) supplemented with 0.6% sodium lactate (BHIL), or on BHIL agar plates. All bacterial strains were grown anaerobically (85% N_2_, 10% CO_2_, 5% H_2_) at 37°C. *Escherichia coli* DH5α cells (Thermo Fisher Scientific) were grown in Luria-Bertani (LB; Difco) medium with aeration at 37°C. *E*. *coli* strains carrying plasmids were grown in LB medium containing spectinomycin (250 μg/ml) or tetracycline (10 μg/ml).

**Table 1:** Bacterial strains and plasmids used in this study.

Strains and plasmids	Characteristics	Reference
Strains		
*E*. *coli* DH5a	Cloning strain	
*V*. *atypica* OK5	Wild type	[9]
*V*. *parvula* PK1910	Wild type	This work
*V*. *parvula* OK1	Wild type	This work
*V*. *atypica* OK2	Wild type	This work
*V*. *rogosae* OK3	Wild type	This work
*V*. *dispar* OK4	Wild type	This work
*V*. *atypica* OK6	Wild type	This work
*V*. *atypica* OK7-1	Wild type	This work
*V*. *dispar* OK7-2	Wild type	This work
*V*. *dispar* OK8	Wild type	This work
*V*. *dispar* OK9	Wild type	This work
*V*. *dispar* OK10	Wild type	This work
*V*. *rogosae* OK11	Wild type	This work
*S*. *gordonii* DL1	Wild type	[10]
*Δhsa*	DL1 *hsa* deletion mutant	This work
*ΔsrtA*	DL1 *srtA* deletion mutant	[11]
*ΔsspA/B*	DL1 *sspA/B* deletion mutant	This work
*S*. *gordonii-hsa*::luc	DL1 *hsa* promoter luciferase reporter	This work
*Δhsa-hsa*::luc	*hsa* promoter luciferase reporter in *hsa* mutant	This work
Plasmids		
pFW5-*hsa*_p_-luc	Suicide vector pFW5::luc of *S*. *gordonii* with *hsa* promoter region; Spec^r^	This work
pAS8741	Plasmid containing the complete *hsa* gene	[12]
pAS8748	Deletion of SR1 and NR2 regions in Hsa	[12]
pAS8744	Deletion of SR1 region in Hsa	[12]
pAS8746	Deletion of NR2 region in Hsa	[12]
pAS8749	Deletion of part of SR2 region in Hsa	[12]
pAS8375	Deletion of part of SR2 region in Hsa	[12]
pAS8165	Deletion of SR2 region in Hsa	[12]
pAS8164	Deletion of SR1 and SR2 regions in Hsa	[12]

### Construction of *S*. *gordonii* surface protein mutants

PCR primers used in this study are listed in Table 2. The *hsa* mutant, *srtA* mutant, and *sspA/B* double mutant were constructed by overlapping PCR. Briefly, the upstream and downstream fragments of the target genes were generated by PCR using the specific primer pairs (Table 2). The *ermAM* fragment was generated by PCR using primer pair *ermAM*-F/*ermAM*-R. The three fragments were mixed and used as template for overlapping PCR without primers. The final amplicons were transformed into *S*. *gordonii* DL1 and mutants were selected for erythromycin resistance.

**Table 2:** Primers used in this study.

Primer	Sequence (5’ to 3’)	Purpose
*hsa*-up-F	TAACAAGCTTAGTACACGGG	*hsa* deletion
*hsa*-up-R	CAAGAATAAACTGCCAAAGCGCGTAACACGTTCAACTTC	*hsa* deletion
*hsa*-dn-F	TATACTACTGACAGCTTCATAGGAGCTTTGGGATTGGC	*hsa* deletion
*hsa*-dn-R	GCATCCCCAAGTTCTGTTTC	*hsa* deletion
*sspAB*-up-F	TCGGTGGTGGGATGACTTAC	*sspA/B* deletion
*sspAB*-up-R	CAAGAATAAACTGCCAAAGTGCCCCTAAGACAGCTCCAC	*sspA/B* deletion
*sspAB*-dn-F	TATACTACTGACAGCTTCATGCCTTACCTAGGCTTGGC	*sspA/B* deletion
*sspAB*-dn-R	CCTACTGTCGGAACTGTACC	*sspA/B* deletion
*srtA*-up-F	CATGGCCTGTAGCTCAATC	*srtA* deletion
*srtA*-up-R	CAAGAATAAACTGCCAAAGGGAAGGAAGCATAAGTTTAATGC	*srtA* deletion
*srtA*-dn-F	TATACTACTGACAGCTTCCCTTCTCGTCTTGCAACTC	*srtA* deletion
*srtA*-dn-R	ACCTAAGAGACGGTGACCAG	*srtA* deletion
*ermAM*-F	CTTTGGCAGTTTATTCTTGAC	gene deletion
*ermAM*-R	GAAGCTGTCAGTAGTATACC	gene deletion
*hsa*_p_-luc-F	ACGCGTCGACACGAAAAGGAGTCCTAGTAGCTACA	Luciferase reportor construction
*hsa*_p_-luc-R	CGCGGATCCGTAATCCCCTCTACTTAATTTAATA	Luciferase reportor construction

### Coaggregation assay

Co-aggregation assays were performed as previously described [13] with minor modifications: Mid-log cells were harvested and washed twice with coaggregation buffer (1 mM Tris buffer [pH 8.0], 0.1 mM CaC1_2_, 0.1 mM MgCl_2_, 150 mM NaCl) at room temperature. Then, cells were resuspended in coaggregation buffer and normalized to OD_600_ = 1.2. Equal volumes (0.1ml) of each cell suspension were mixed in a micro-centrifuge tube and vortexed briefly. The tubes were left standing at room temperature until aggregates appeared, usually 15–30 min. Tubes containing each cell suspension alone (0.2 ml) were included as controls. All assays were repeated at least 3 times.

### Construction of *hsa* reporter strain

The *hsa*-luc reporter was constructed as follows: A ~ 600 bp fragment of the *hsa* promoter region was PCR amplified using primer pairs *hsa*_p_-luc-F/*hsa*_p_-luc-R (Table 2). The product was digested with *Sal*I and *Bam*HI and cloned into the suicide vector pFW5-luc digested with the same restriction enzymes to create the fusion plasmid pFW5-*hsa*_p_-luc. The confirmed plasmid was transformed into *S*. *gordonii* DL1 and transformants were selected on BHI plates supplemented with spectinomycin (500 μg mL^-1^). Transformants were further confirmed by PCR.

### Luciferase assays

Overnight culture of *S*. *gordonii hsa*-luc reporter strains were spun down and re-suspended with fresh BHI media to OD_600_ ~1.0. Suspended cultures then were 1:20 diluted into fresh BHI media. For co-culture test, overnight culture of *V*. *atypica* OK5 was centrifuged and re-suspended with fresh BHI to OD_600_ ~1.0, and then was diluted into BHI media with *S*. *gordonii/V*. *atypica* ratio 5:1. All cultures were grown in anaerobic condition for 24 hours, and the data were measured every 2 hours. Luciferase assays were performed by adding 25 μL of 1 mM D-luciferin (Sigma) solution (suspended in 0.1 M citrate buffer, pH 6.0) into 100 μL samples, and luciferase activities were measured using a TD 20/20 luminometer (Turner Biosystems, Sunnyvale, CA). For single species cultures, the optical densities at 600 nm were measured with a spectrophotometer (Bio-Rad, smartspec 3000). For mixed species cultures, colony-forming units (CFU/ml) were obtained by plate counting. Luciferase activity was expressed either as relative light unit (RLU)/OD_600_ (for single-species culture), or as RLU/CFUx1000 (for mixed species culture).

## Results

### Coaggregation between *S*. *gordonii* DL1 and veillonellae

Coaggregation between oral streptococci especially *S*. *gordonii* and veillonellae has been studied previously [8, 14]. To gain a general picture of the coaggregation pattern between our model strain *S*. *gordonii* DL1 and our clinical veillonellae isolates, we used the modified *in vitro* coaggregation assay [13] to qualitatively assess the coaggregation ability between *S*. *gordonii* DL1 and the veillonellae strains. As shown in Table 3, *S*. *gordonii* DL1 coaggregated with two of four *V*. *atypica* strains, all two *V*. *parvula* strains, and one of two *V*. *rogosae* strains. Interestingly, none of the five *V*. *dispar* strains coaggregated with *S*. *gordonii* DL1.

**Table 3:** Coaggregation of *S*. *gordonii* wild-type and *Δhsa* strain with various *Veilloenlla* species/ strains.

Strain name	*S*. *gordonii* DL1	*Δhsa*
***V*. *atypica* OK2**	**+**	**−**
***V*. *atypica* OK5**	**+**	**−**
***V*. *atypica* OK6**	**−**	**NT**
***V*. *atypica* OK7-1**	**−**	**NT**
***V*. *dispar* OK7-2**	**−**	**NT**
***V*. *dispar* OK8**	**−**	**NT**
***V*. *dispar* OK9**	**−**	**NT**
***V*. *dispar* OK10**	**−**	**NT**
***V*. *dispar* OK4**	**−**	**NT**
***V*. *parvula* PK1910**	**+**	**+**
***V*. *parvula* OK1**	**+**	**−**
***V*. *rogosae* OK3**	**+**	**−**
***V*. *rogosae* OK11**	**−**	**NT**

+: coaggregation; -: no coaggregation; NT: Not Tested

### Identification of the adhesin in *S*. *gordonii* DL1 responsible for coaggregation with veillonellae

**Fig 1:**
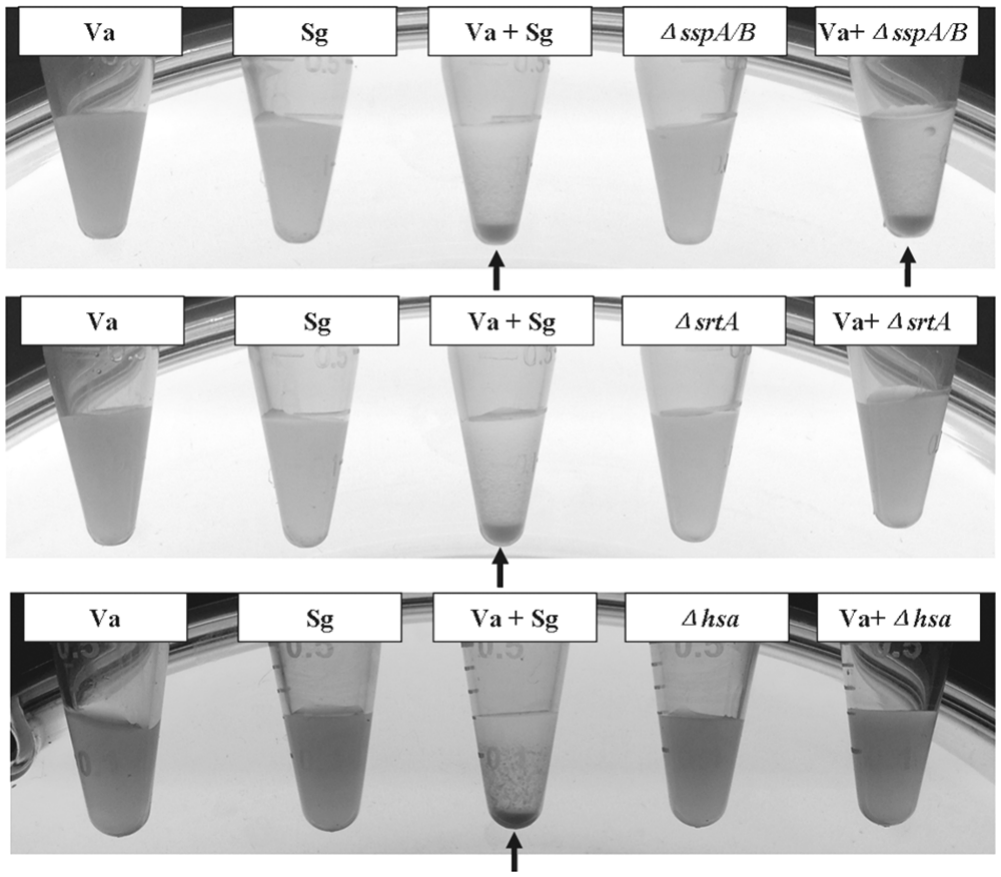
Coaggregation of *S*. *gordonii* DL1 (wt) and surface protein mutants with *V*. *atypica* OK5. Coaggregation-positive pairs form aggregates that precipitate at the bottom of the tube (arrowhead), while cells of the single species cultures or mixed cultures with the *hsa* and *srt*A mutants remain in suspension. Sg: *S*. *gordonii* DL1; Va: *V*. *atypica* OK5.

In our previous study, we demonstrated that proteinase K treatments of *S*. *gordonii* abolished co-aggregation with *V*. *atypica*, suggesting that a surface protein/adhesin is probably involved [8]. Four types of surface adhesins have been identified in *S*. *gordonii*: cell-surface fibrillar proteins (CshA and CshB), sialic-acid binding protein (Hsa/GspB), amylase-binding proteins (AbpA and AbpB), and antigen I/II (AgI/II) family proteins (SspA and SspB) [15–19]. To have a quick survey of the known surface adhesins in *S*. *gordonii*, we initially constructed mutant strains with deletion of surface adhesin genes *sspA/B*, *hsa*, and *srtA*. As both Hsa and SspA/B belong to LPXTG-motif-containing surface proteins, SrtA was used as a positive control because its deletion would eliminate all surface proteins with a LPXTG-containing cell wall anchoring domain [11]. In vitro coaggregation assay was used to assess the effect of these mutations on coaggregation with *V*. *atypica*. As shown in Fig 1, the *sspA/B* double mutation did not have any effect on coaggregation with *V*. *atypica*, while the *hsa* and *srtA* mutations completely abolished coaggregation. This result suggested that Hsa is the protein responsible for coaggregation with *V*. *atypica*. To see if the *hsa* mutation had the same effect on other *Veillonella* strains that coaggregated with *S*. *gordonii*, the same coaggregation assay was performed. As shown in Table 3, the mutation abolished coaggregation with all but one (*V*. *parvula* PK1910) coaggregation partner. This result indicates that Hsa is the adhesin mediating coaggregation with *V*. *atypica* strains OK2 and OK5, *V*. *parvula* strain OK1, and *V*. *rogosae* OK3, and that coaggregation with *V*. *parvula* PK1910 (previously *V*. *atypica* PK1910) is probably mediated by a completely different mechanism.

### Identification of Hsa domains that are responsible for coaggregation between *S*. *gordonii* DL1 and *V*. *atypica* OK5

**Fig 2:**
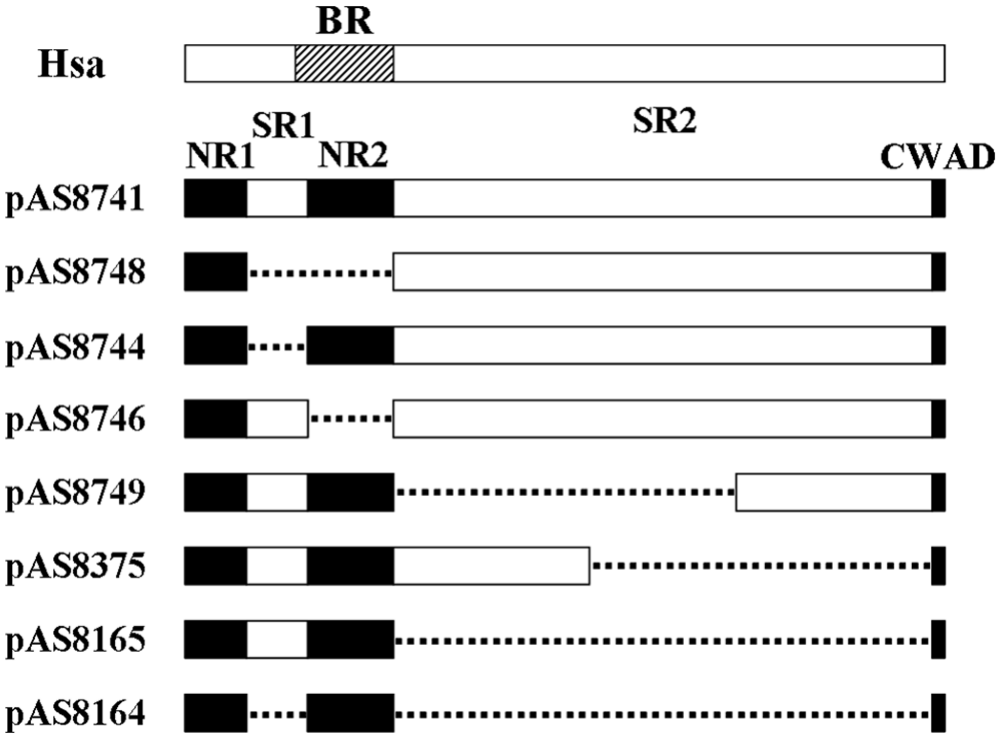
Graphic illustration of the various regions deleted in the complementing plasmids. **The basic region of Hsa was located on amino acid position 228–449.** pAS8741 carries an intact *hsa* gene; pAS8748 encodes a Hsa protein lacking the SR1 and NR2 regions (Hsa_Δ105–446_); pAS8744 encodes a Hsa protein lacking the SR1 region (Hsa_Δ105–245_); pAS8746 encodes a Hsa protein lacking the NR2 region (Hsa_Δ246–446_); pAS8749 encodes a Hsa protein lacking the N-terminal part of the SR2 region (Hsa_Δ448–1459_); pAS8375 encodes a Hsa protein lacking the C-terminal part of the SR2 region (Hsa_Δ1092–2143_); pAS8165 encodes a Hsa protein lacking the entire SR2 region (Hsa_Δ448–2143_); and pAS8164 encodes a Hsa protein lacking both SR1 and SR2 regions (Hsa_Δ105–245, Δ448–2143_). The dotted lines indicate the regions deleted. BR: basic region; NR: non-repeat region; SR: serine-rich repeat region; CWAD: cell wall anchoring domain.

**Fig 3:**
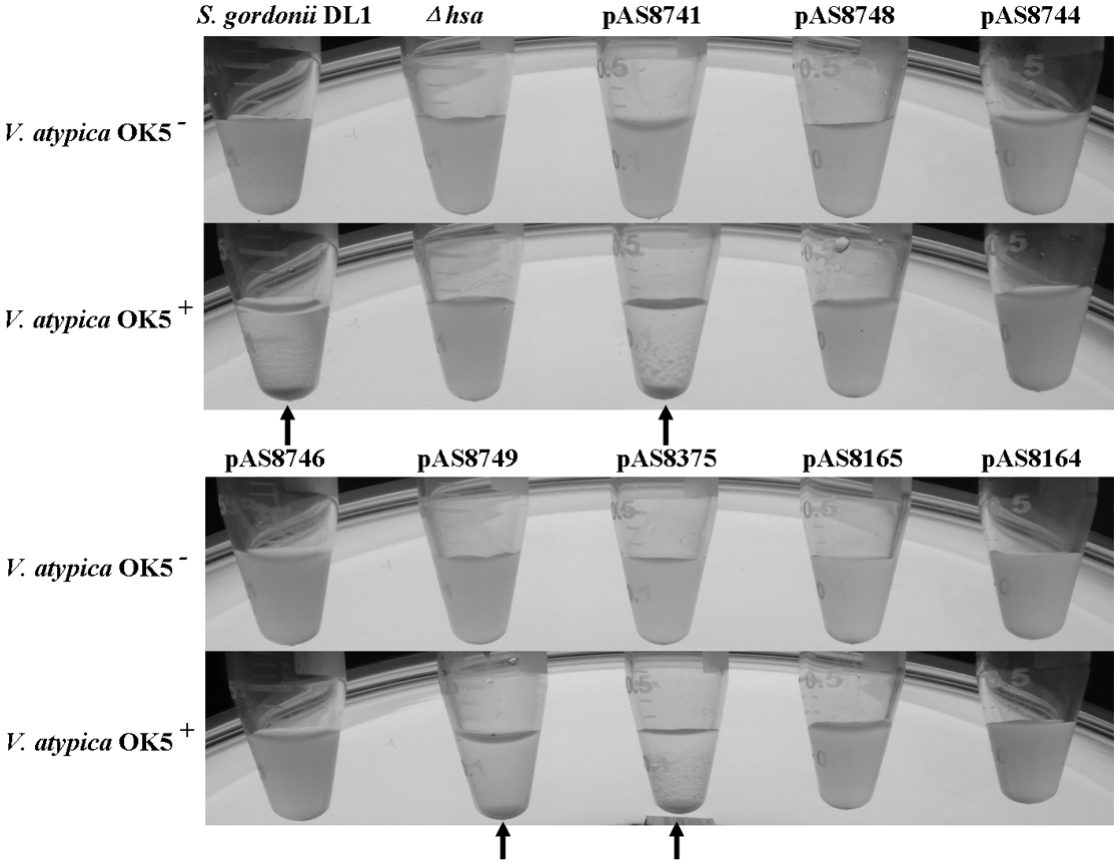
Coaggregation of *S*. *gordonii* DL1 (wt), *hsa* mutant (*Δhsa*), and the eight complemented strains with *V*. *atypica* OK5. *S*. *gordonii* DL1 and complemented strains containing pAS8741, pAS8749 and pAS8375 formed aggregates with *V*. *atypica* OK5 and precipitated at the bottom of the tube (arrowhead), while cells of the single species cultures or mixed cultures with the *Δhsa* mutant or complemented strains containing pAS8748, pAS8744, pAS8746, pAS8165 and pAS8164 remained in solution.

The surface adhesin Hsa/GspB has been characterized extensively [16, 19]. The protein has been shown to bind to sialic acid on the surface of different types of mammalian cells [20–22]. In *S*. *gordonii*, the protein has 2178 amino acids, which can be divided into 5 domains: the N-terminal non-repeat region (NR1), followed by serine-rich repeat region 1 (SR1), non-repeat region 2 (NR2), serine-rich repeat region 2 (SR2), and the C-terminal wall-anchor domain (CWAD) [Fig 2, [12]]. A series of plasmids containing deletions of different domains has been previously constructed by Dr. Takahashi’s group, and all plasmids were shown by Western blot to express truncated Hsa proteins corresponding to the respective *hsa* deletions they carry in the mutant strains [12]. To determine which domain is responsible for binding to *V*. *atypica*, we used these plasmids to complement the *hsa* deletion mutation strain. A total of 8 complement strains were thus constructed and tested in an *in vitro* coaggregation assay with *V*. *atypica*. As shown in Fig 3, five strains containing plasmids pAS8748, pAS8744, pAS8746, pAS8165, and pAS8164 failed to restore coaggregation with *V*. *atypica*, suggesting that the deleted regions of Hsa encoded by the DNA fragments in these plasmids are required for coaggregation. As illustrated in Fig 2, plasmid pAS8165 contains a complete deletion of the SR2 region, suggesting that the SR2 region is required by coaggregation. However, since plasmids pAS8749 contains only the C-terminal part of SR2, and plasmid pAS8375 contains only the N-terminal part of SR2, and both restored coaggregation with *V*. *atypica*, it appears that the N- and C-terminal region of SR2 is redundant for Hsa function. In addition to the SR2 fragment, both SR1 and NR2 domains are also required for coaggregation, because plasmids containing SR1 (pAS8746) or NR2 (pAS8744) deletions did not complement the coaggregation deficiency of the *hsa* deletion. Taken together, we conclude that the intact domains of SR1 and NR2 plus either the N- or C-terminal domain of SR2 are required for Hsa binding to *V*. *atypica*.

### Sialic acid is not involved in Hsa binding to *V*. *atypica* OK5

**Fig 4:**
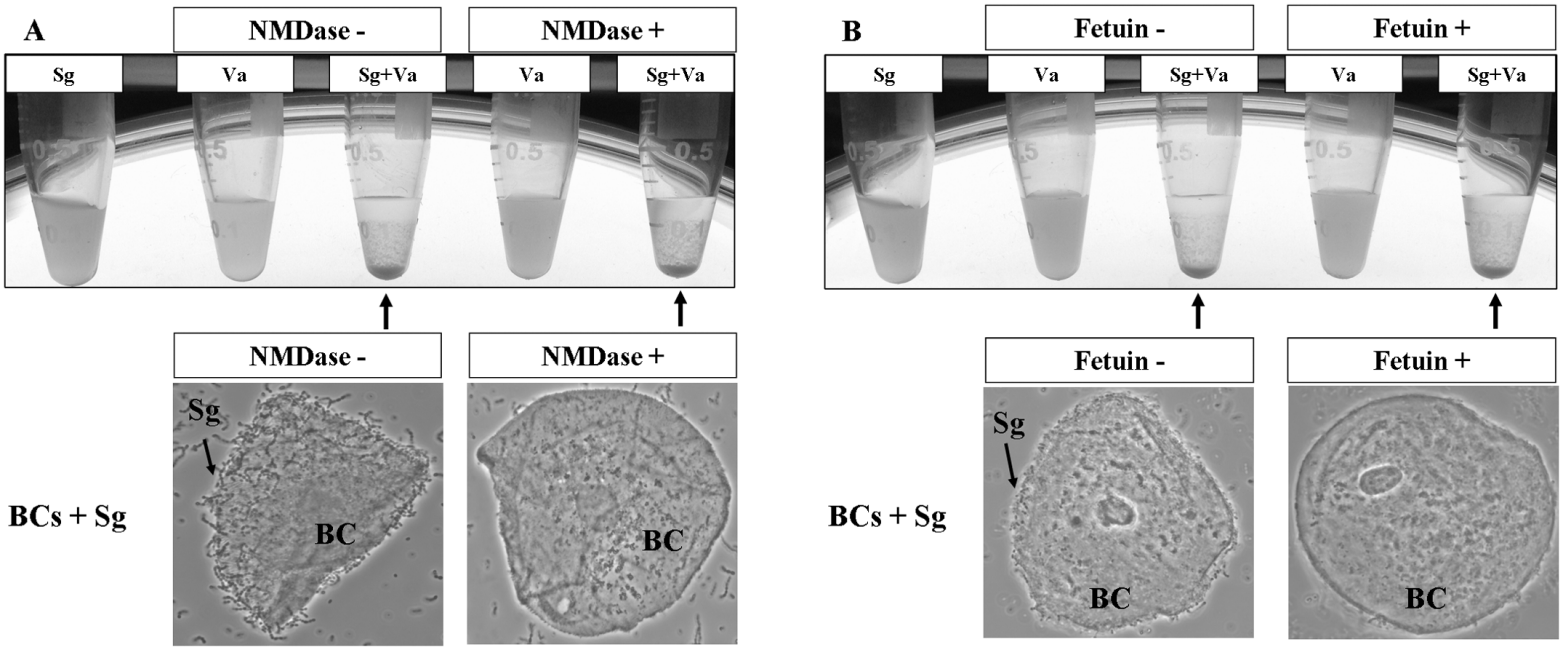
Coaggregation of *S*. *gordonii* DL1 with *V*. *atypica* OK5 treated with neuraminidase (A) or fetuin (B). Note that none of the treatments affected coaggregation between the two species, and *S*. *gordonii* cannot adhere to buccal cells treated by neuraminidase or fetuin. The arrows indicate the aggregates at the bottom of the tube. Sg: *S*. *gordonii* DL1; Va: *V*. *atypica* OK5.

It was well known that sialic acid is the binding partner for Hsa adhesin, and the basic region (BR) of Hsa mediates its adhesion to sialic acid[23, 24]. The BR of Hsa is located on amino acid position 228–449, encompassing the C-terminal portion of SR1 and the entire region of NR2 (Fig 2). Results presented above demonstrated that both SR1 and NR2 regions are also required for coaggregation with *V*. *atypica* OK5, suggesting that sialic acid or similar molecules may exist on the veillonellae cell surface[20, 25]. To see whether *V*. *atypica* could potentially synthesize sialic acids, we searched the genome sequence of *V*. *atypica* strains in the HOMD database for putative sialic acid biosynthesis genes, and found none. The draft sequence of strain *V*. *atypica* OK5 (P. Zhou and F. Qi, unpublished data) used in this study was also searched, and no sialic acid biosynthesis genes were found. To further confirm that no sialic acid from the *V*. *atypica* cell surface is involved in coaggregation with *S*. *gordonii*, we first treated *V*. *atypica* OK5 with neuraminidase prior to the coaggregation assay. This treatment did not affect coaggregation, although similar treatment of human buccal cells completely abolished attachment by *S*. *gordonii* DL1 (Fig 4A). Next, we added fetuin to the coaggregation assay. Fetuin is a group of molecules with sialylated carbohydrate structures that are similar to the sialoproteins on mammalian cell surface [23]. We reasoned that if a sialic acid-like structure on *V*. *atypica* cell surface were involved, then adding fetuin would inhibit coaggregation. It turned out that fetuin had no effect on coaggregation either (Fig 4B). Similarly, adding fetuin to the buccal cells nearly completely abolished attachment by *S*. *gordonii* DL1 cells (Fig 4). Taken together, we concluded that sialic acid is not involved in Hsa-mediated coaggregation with *V*. *atypica* OK5, although it is required for *S*. *gordonii* attachment to human buccal cells.

### The expression of *hsa* gene is not regulated by coaggregation with *V*. *atypica*

**Fig 5:**
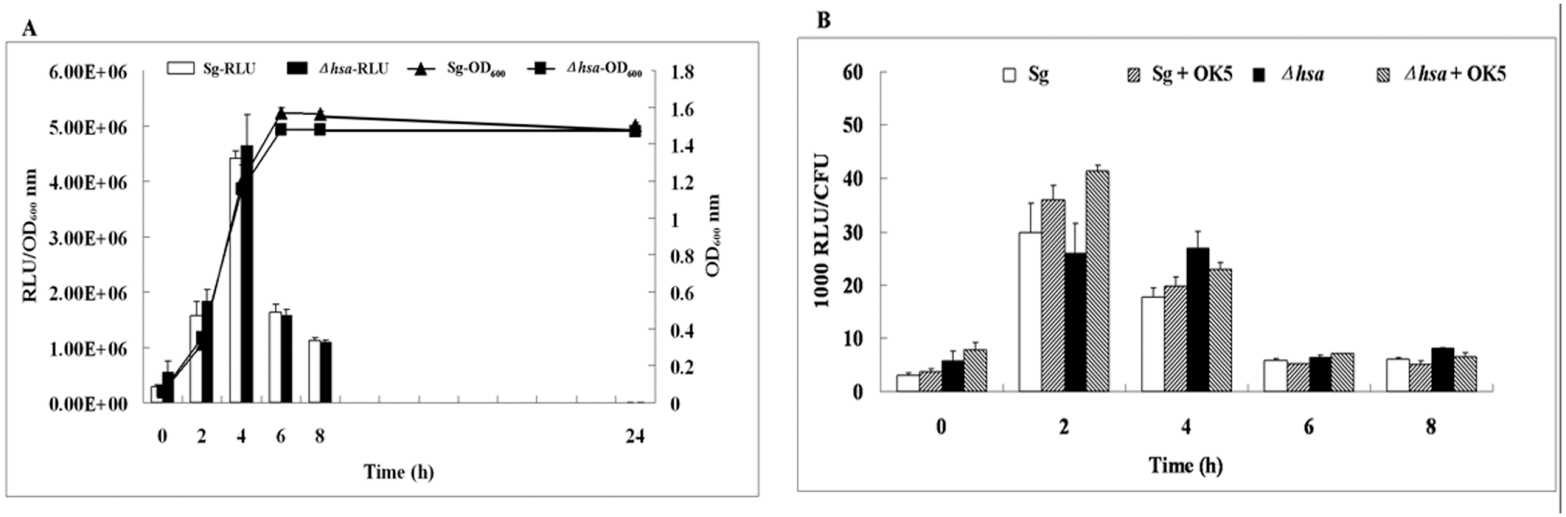
**(A) Luciferase expression pattern and growth curve of *S*. *gordonii* DL1 and *Δhsa* mutant.** The growth of 2 strains was measured every 2 hours and at OD_600_ nm, and luciferase expression (relative light unit, RLU) was normalized by growth curve (OD_600_). (B) Luciferase expression pattern of *S*. *gordonii* DL1 and *Δhsa* mutant mixed with *V*. *atypica* OK5. CFU (colony-forming unit) was measured every 2 hours and utilized to normalize RLU. 1000 RLU/CFU was shown in Y-axis. Sg: *S*. *gordonii* DL1; OK5: *V*. *atypica* OK5. The results are shown as the means ± SD of three independent experiments.

To see whether the expression of *hsa* gene is regulated by coaggregation between *S*. *gordonii* and *V*. *atypica*, we constructed a firefly luciferase reporter in the *S*. *gordonii* DL1 wild type as well as in the *Δhsa* mutant strain. The expression of *hsa*-luc reporter was first measured over the growth curve in the wild type and the mutant single species cultures. As shown in Fig 5A, the *hsa* gene expression started at early log phase, peaked at mid-log phase, and rapidly turned down at early stationary phase. There is no difference in the level or the pattern of gene expression in the wild-type and the *hsa* mutant strains, suggesting no feedback regulation. Next, we measured *hsa*-luc expression of the two strains in mixed cultures with *V*. *atypica* OK5 (Fig 5B). No difference was observed between single and mixed species cultures, or between coaggregated (*S*. *gordonii* wild-type + OK5) and non-coaggregated (*S*. *gordonii Δhsa* + OK5) mixed cultures, suggesting that coaggregation does not affect gene expression of *hsa*.

## Discussion

In the oral biofilm, cell-cell coaggregation plays a critical role in biofilm development. Of particular importance is the coaggregation between pioneer colonizers and the early/middle colonizers. As the latter are bridging species for colonization of late colonizers many of which are periodontopathogens, understanding the mechanisms of coaggregation between the pioneer colonizers and the bridging species can lead to development of knowledge-based strategy for prevention of periodontal diseases.

The mitis streptococci are major pioneer colonizers, and species of the *Veillonella* genus are one of the most prevalent and numerically dominant bridging species. The two groups of bacteria physically coaggregate with each other especially amongst strains isolated from the same anatomical sites [14, 26]. This coaggregation enables the *Veillonella* species to join the early biofilm community, for the veillonellae bacteria on their own have very weak (if any) attachment ability to the salivary pellicles on the tooth’s surface [1, 27, 28].

A number of early studies attempted to identify surface adhesins mediating coaggregation between streptococci and veillonellae [29, 30]. Weerkamp and McBride analyzed the surface component that is responsible for *Streptococcus salivarius* HB binding to *V*. *alcalescens* V1 (now *V*. *parvula* V1), and they found that a fraction adhered to *V*. *alcalescens* V1 was released upon lysozyme treatment of the latter [29]. Using chemical mutagenesis and natural selection, Handley et al. selected four mutants of *S*. *salivarius* HB that abolished the ability to coaggregate with *V*. *parvula* V1. However, the nature of these mutants has not been determined [29, 30]. Thus, until now no adhesin has been definitively identified in streptococci that is responsible for coaggregation with veillonellae. In this study, we identified Hsa from *S*. *gordonii* as the adhesin mediating coaggregation with *V*. *atypica* strains OK2 and OK5, *V*. *rogosae* strain OK3, and *V*. *parvula* strain OK1, but not with *V*. *parvula* strain PK1910 (Table 3), Additionally, not all strains in *V*. *atypica* and *V*. *rogosae* coaggregated with *S*. *gordonii*, suggesting that this intergeneric coaggregation may be strain specific, which is also consistent with previous reports [26].

The finding that Hsa being the adhesin mediating coaggregation of *S*. *gordonii* with some *Veillonella* species/strains is very interesting, because Hsa has been well characterized as a sialic acid binding adhesin mediating attachment of *S*. *gordonii* to human cells including heart tissues [31]. Thus, Hsa has been considered an important virulence factor in infective endocarditis [32, 33]. Our finding demonstrates, for the first time, that Hsa is perhaps a multivalent adhesin mediating not only attachment to mammalian cells but also to prokaryotic cells. Interestingly, in our recent study, we identified a surface adhesin, Hag1, in *V*. *atypica*, which mediates coaggregation not only with several bacterial species including *S*. *gordonii*, but also with human buccal cells [8]. Whether it is common for oral microbial species to produce adhesins with binding capacity to multiple partners awaits further investigation.

Perhaps the most exciting finding from this study is that the same functional domains of Hsa required for binding to sialic acid on mammalian cells are also required for binding to *Veillonella* cells. However, genomic analysis, combined with neuraminidase treatment of *V*. *atypica* cells and fetuin inhibition assay, failed to provide evidence for the existence of sialic acids or similarly structured molecules on the *Veillonella* cell surface. Thus, there exists a possibility that subdomains may exist in the binding domain of Hsa, which could be responsible for binding to different target molecules on different cell surfaces. Detailed genetic mutagenesis and identification of the amino acid residues involved in binding are needed to answer this question.
